# Higher groundwater levels in western Europe characterize warm periods in the Common Era

**DOI:** 10.1038/s41598-020-73383-8

**Published:** 2020-10-01

**Authors:** Willy Tegel, Andrea Seim, Georgios Skiadaresis, Fredrik Charpentier Ljungqvist, Hans-Peter Kahle, Alexander Land, Bernhard Muigg, Kurt Nicolussi, Ulf Büntgen

**Affiliations:** 1 Chair of Forest Growth and Dendroecology, Institute of Forest Sciences, Albert-Ludwigs-University Freiburg, 79106 Freiburg, Germany; 2 Department of Botany, University of Innsbruck, 6020 Innsbruck, Austria; 3 Chair of Silviculture, Institute of Forest Sciences, Albert-Ludwigs-University Freiburg, 79106 Freiburg, Germany; 4 Department of History, Stockholm University, 106 91 Stockholm, Sweden; 5 Bolin Centre for Climate Research, Stockholm University, 106 91 Stockholm, Sweden; 6 Swedish Collegium for Advanced Study, 752 38 Uppsala, Sweden; 7 Institute of Biology, University of Hohenheim, 70599 Stuttgart, Germany; 8 University of Applied Forest Sciences, Schadenweilerhof, 72108 Rottenburg am Neckar, Germany; 9 Chair of Forest History, Institute of Forest Sciences, Albert-Ludwigs-University Freiburg, 79106 Freiburg, Germany; 10 Institute of Geography, University of Innsbruck, 6020 Innsbruck, Austria; 11 Department of Geography, University of Cambridge, Cambridge, CB2 3EN UK; 12 Swiss Federal Research Institute (WSL), 8903 Birmensdorf, Switzerland; 13 Global Change Research Centre (CzechGlobe), 61300 Brno, Czech Republic

**Keywords:** Climate sciences, Environmental sciences, Hydrology

## Abstract

Hydroclimate, the interplay of moisture supply and evaporative demand, is essential for ecological and agricultural systems. The understanding of long-term hydroclimate changes is, however, limited because instrumental measurements are inadequate in length to capture the full range of precipitation and temperature variability and by the uneven distribution of high-resolution proxy records in space and time. Here, we present a tree-ring-based reconstruction of interannual to centennial-scale groundwater level (GWL) fluctuations for south-western Germany and north-eastern France. Continuously covering the period of 265–2017 CE, our new record from the Upper Rhine Valley shows that the warm periods during late Roman, medieval and recent times were characterized by higher GWLs. Lower GWLs were found during the cold periods of the Late Antique Little Ice Age (LALIA; 536 to ~ 660 CE) and the Little Ice Age (LIA; between medieval and recent warming). The reconstructed GWL fluctuations are in agreement with multidecadal North Atlantic climate variability derived from independent proxies. Warm and wet hydroclimate conditions are found during warm states of the Atlantic Ocean and positive phases of the North Atlantic Oscillation on decadal scales.

## Introduction

**Figure 1:**
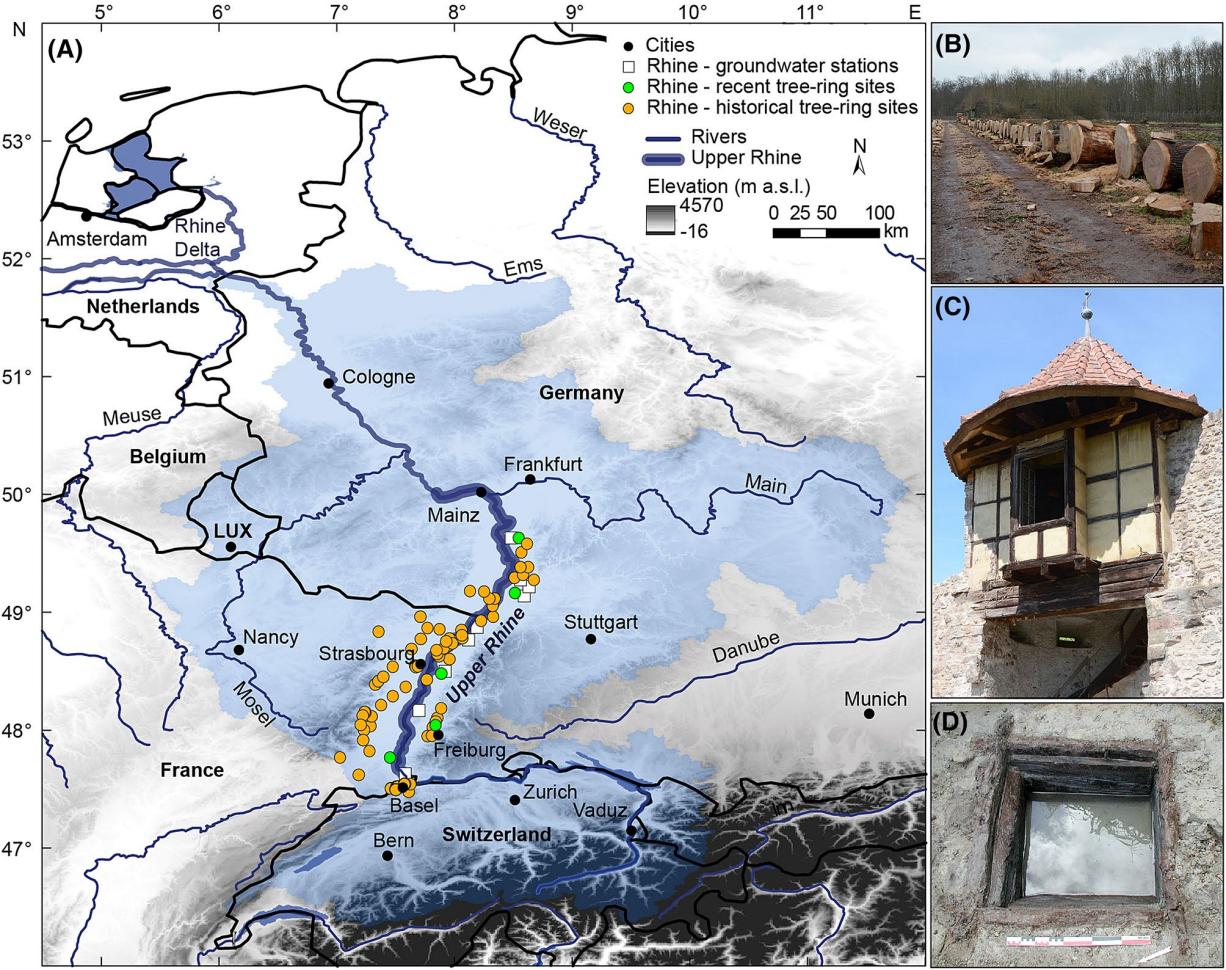
**(A)** Catchment area of the river Rhine (light blue) with location of sites of historical (orange dots) and recent (green dots) oak (*Quercus *spp.) samples and groundwater monitoring stations (white squares) in the Upper Rhine Valley. Examples of sources for (**B)** recent oak trees, (**C)** historical timbers and (**D)** archaeological timbers. The map reflects knowledge from the authors and https://www.eea.europa.eu/data-and-maps/data/european-catchments-and-rivers-network and was created via software ArcGIS 10.6 by Esri.

**Figure 2:**
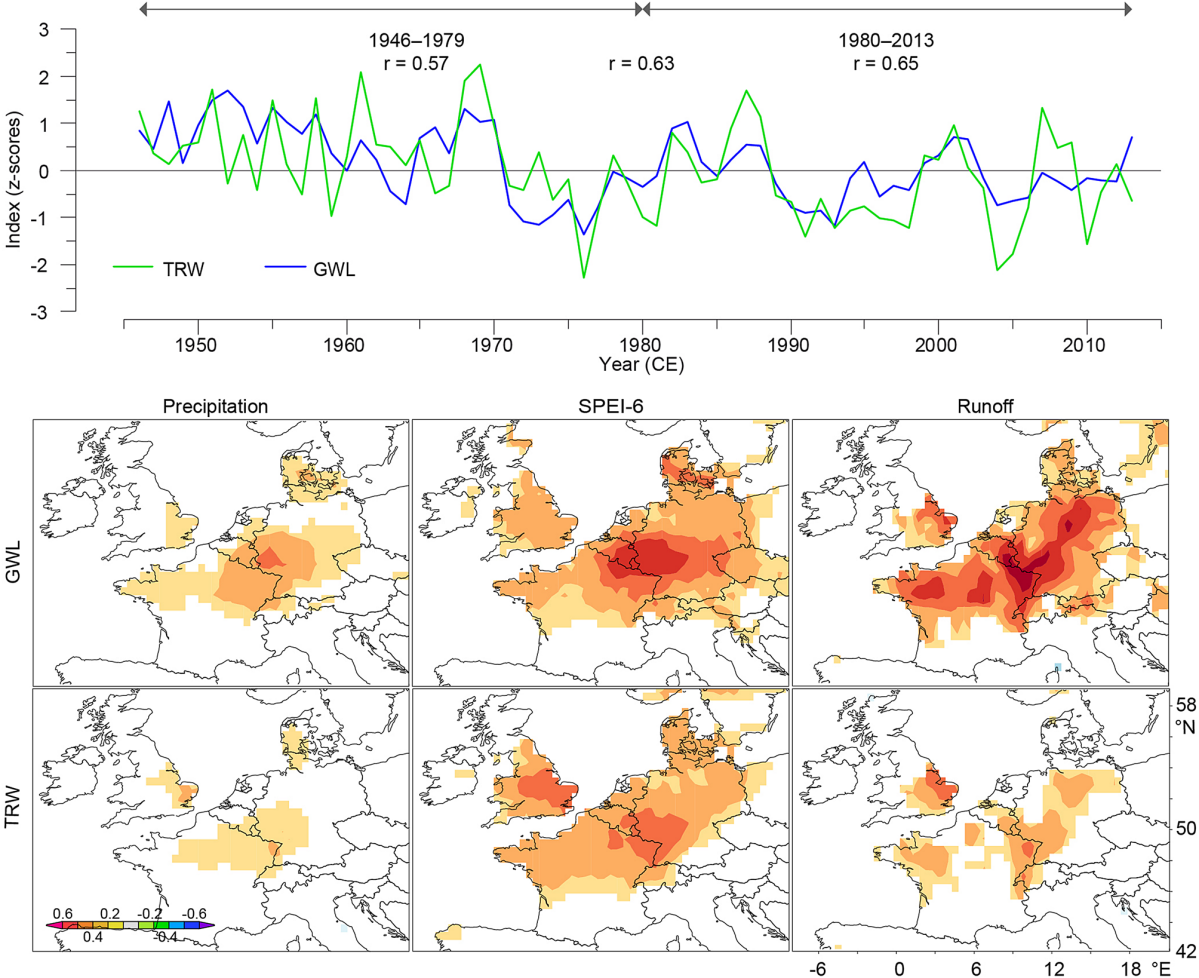
The Upper Rhine Valley (7°E–8.5°E and 47.5°N –50°N) annual groundwater level (GWL) variability (Fig. 1, S2 and Tab. S2) and tree-ring width (TRW) data after Regional Curve Standardisation (RCS) detrending with correlation results for the period 1946–2013 and two sub-periods. Spatial correlation maps between (above) annual GWL and (below) RCS detrended TRW data and hydroclimate variables of precipitation, SPEI-6 and runoff using CRU TS4.03 climate grid data (0.5° × 0.5°, *p* < 0.10) for the period 1946–2013. All maps were created via the KNMI online server (https://climexp.knmi.nl).

The majority of the world’s population lives in river valleys, where groundwater access is crucial not only for sustaining the function and productivity of natural and agricultural systems but also for human well-being. With 45 billion m^3^, the Upper Rhine Plain is among the largest aquifers in Europe, supplying more than three million people with freshwater (Fig. 1). Today, the Rhine Valley is one of the most ecologically and economically important regions in Europe, stretching over six countries with a length of 1230 km. In view of global warming, where accelerated hydrological processes may contribute to intense and severe hydroclimate extremes such as droughts and floods, it is indispensable to understand how anthropogenic influences superimpose upon natural hydroclimate variability. The relative effects of temperature on hydrological cycles remain ambiguous, with some studies for Western and Central Europe indicating a general drying trend under a warming climate and others predicting wetter conditions^1,2^. A retrospective view of the history of hydroclimate based on proxy data can be a valuable contribution towards a better understanding of the long-term temperature-moisture dependency of the climate system^3–5^. Due to their explicit spatio-temporal resolution and inherent moisture information, the tree-ring width (TRW) data provides the most suitable proxy archives for reconstructing hydroclimate^6,7^. The close relationships between the groundwater level (GWL), runoff and soil moisture are controlled by temperature and precipitation and reflect the fundamental water balance (Fig. 2 and Supplementary Figs. S3 and S7)^8,9^. In contrast to precipitation, GWL fluctuations reflect hydrological variations over larger spatial and temporal scales as aquifers store and transmit water. Generally, the high GWL in most parts of the Rhine Plain causes a close co-variation between soil moisture and GWL, which are key variables controlling oak (*Quercus* spp.) growth^10^.

Here, we use 1027 individual TRW series from the Upper Rhine Valley to reconstruct long-term GWL variations and to identify annual GWL extremes for the period of 265–2017 CE. Based on the new reconstruction, we contribute to the current discussion of the extent to which dry and wet conditions occurred during cold and warm periods in the Common Era.

### Reconstructing GWL fluctuations over the Common Era

To better understand past hydroclimatic conditions, we developed a moisture-sensitive oak TRW chronology for the Upper Rhine Valley in south-western Germany and north-eastern France. The chronology covers the period of 265–2017 CE, consisting of 752 archaeological, subfossil and historical samples (henceforth called historical) collected at 127 sites and 285 samples from living trees from five sites (Fig. 1 and Supplementary Figs. S1 and S2). The different temporal assemblages of the samples with distinct growth characteristics required horizontal splitting of the entire dataset into two historical subsets (hist 1 and hist 2) and a recent subset (Supplementary Fig. S2) prior to age-related growth trend elimination (Supplementary Figs. S1 and S2A,B)^11^.

The number of samples varied considerably over time. It is highest, with 285 TRW series, from the 1960s–1980s and lowest, with 16 TRW series, from 1145–1149 CE. This minimum might be associated with the historical settlement boom and urban development during the thirteenth century, which led to the replacement of many constructions from the eleventh and twelfth centuries CE^12^. Quality measures of the chronology, such as the common signal strength (so-called expressed population signal (EPS)) and inter-series correlation (Rbar), are high throughout the chronology (Supplementary Fig. S2D,E), and a short-term drop in values below the commonly applied EPS threshold of 0.85 corresponds to the period of lowest replication (Supplementary Fig. S2E). Our new oak chronology retaining low-frequency variations preserved using the Regional Curve Standardisation (RCS) detrending procedure (see “Methods”) shows a significant positive correlation (*r* = 0.63, *p* < 0.001) with the mean annual GWL fluctuations in the Upper Rhine over the well-replicated period of 1946–2013 CE (Figs. 1A and 2 and Supplementary Table S1). The annual GWL fluctuations and oak growth variability are strongly related to the local hydroclimatic variability, which is reflected by the strong positive correlations observed mainly for SPEI-6 and runoff (Fig. 2). These are spatially most pronounced in the Upper Rhine Valley but extend to almost all of Western and Central Europe (Fig. 2). The stable GWL-oak growth relationship (Fig. 2, see “Methods”) allowed for the development of a GWL reconstruction that covers the period of 265–2017 CE and represents hydroclimate variability in Western and Central Europe, containing both long-term GWL variations and the occurrence of extreme events in the high-frequency domain.

### GWL variability and its forcing

**Figure 3:**
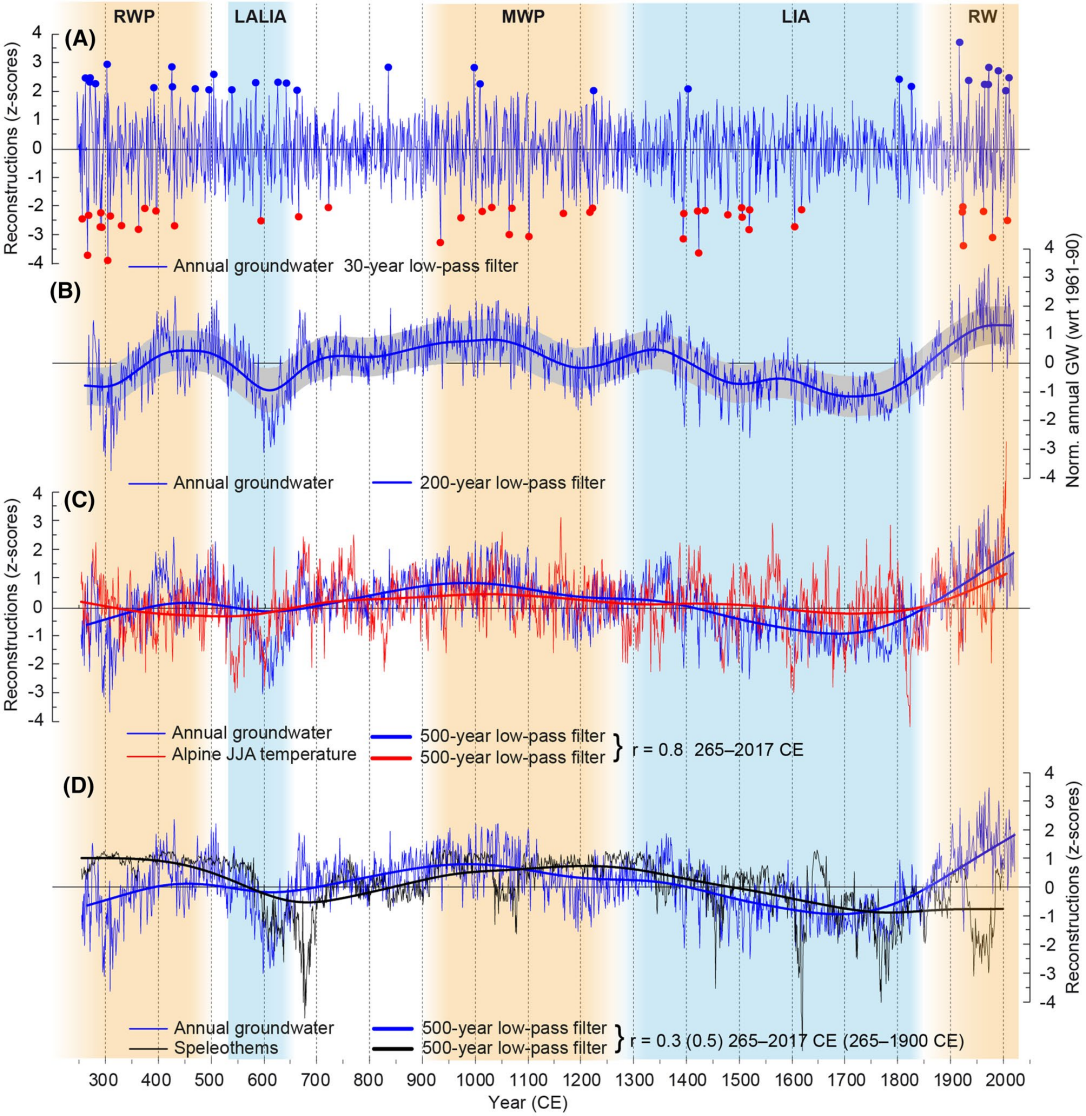
The new 265–2017 CE groundwater reconstruction for the Upper Rhine Valley, with colder and warmer climate periods highlighted (*RWP* Roman Warm Period, *LALIA* Late Antiquity Little Ice Age, *MWP* Medieval Warm Period, *LIA* Little Ice Age, *RW* Recent Warming). (**A)** High-frequency annual GWL reconstruction with low (red dots) and high (blue dots) extreme years highlighted which are defined by ± 2 standard deviation. (**B)** Reconstructed annual GWL with preserved long-term trends. The uncertainty range (grey shading) is measured by the single root mean square error combined with the EPS value of the calibration period. Comparison of our annual GWL reconstruction for the Upper Rhine Valley [correlation coefficients (r) in legend] and (**C)** the Austrian Alps June–August temperature^6^ and (**D)** speleothem record^5^ representing North Atlantic hydroclimate from north-western Scotland over the period 265–2003 CE.

Variations in oak growth in the high-frequency domain reflect considerable alterations in the occurrence of a total of 31 high and 44 low GWL extremes over the 265–2017 CE period (see “Method”, Fig. 3A and Supplementary Fig. S8). The most favourable growing conditions and thus high GWLs were observed in 1914, whereas the most severe growth reduction, due to low GWLs, occurred in 308 CE and in 1921 CE as the most recent example (Fig. 3A and Supplementary Figs. S8–10). Accumulations of extremely high and low GWLs were found during the mid-fourth to eighth century CE and from the beginning of the twentieth century to the present (Fig. 3A). Clusters of groundwater droughts are recorded during the second half of the third century CE and from the beginning of the fifteenth to the sixteenth century CE. Extremes in TRW data document positive or negative growing conditions, and the attribution to causal factors can to a certain extent be complicated by lagged growth responses to climatic conditions prevailing in the year(s) prior to ring formation^13^. For oaks in particular, growth resumption in spring depends highly on carbon reserves stored in the previous year, and thus, annual growth rates might partly reflect climatic conditions in the year prior to ring formation^14^. Moreover, prolonged and enhanced soil moisture availability of late snowmelt and high GWLs can also have a distorting effect that does not reflect the real effect of a dry summer on tree growth^15^. Another possible effect might be that high moisture content in the soil accumulated during late summer and autumn could be carried over the following winter and thus affect cambial activity and carbon uptake early in the growing season^16^. Furthermore, GWL extremes in the past 100 years might reflect increased anthropogenic impacts on groundwater reservoirs through, e.g., increased groundwater extraction rates, rather than natural or climate change-driven extremes^9^. This might explain a noticeable increase in both positive and negative GWL extremes in the modern period (Fig. 3A and Supplementary Fig. S8).

To gain comprehensive insights into the full spectrum of climate variability, we compared our GWL reconstruction with a TRW-based summer temperature reconstruction developed from high-elevation conifers from the Austrian Alps: 1089 Stone pine (*Pinus cembra* L.) and 457 European larch (*Larix decidua* Mill.) samples (Fig. 3C)^6^. The Austrian Alps temperature reconstruction correlates with the groundwater reconstruction over their common period of 265–2003 CE with *r* = 0.17 (*p* < 0.001) and *r* = 0.81 (*p* < 0.001) for the unfiltered and 500-year low-pass filtered versions, respectively.

Consistently, long-term positive anomalies in reconstructed GWLs and summer temperatures can be observed from the end of the seventh century until the mid-fourteenth century CE (Fig. 3C). The highest GWLs are found during the tenth and eleventh centuries CE, which are the two centuries with the strongest medieval warming^17,18^. Conversely, the new GWL reconstruction shows overall relatively low levels at the beginning of the Early Middle Ages, including the Late Antique Little Ice Age (LALIA; 536 to ~ 660 CE) and during the Little Ice Age (LIA) from the late-fourteenth to the late-nineteenth century CE. In addition to below-average temperatures during the LIA, low GWLs were accompanied by intense agriculture and forest management practices, including deforestation in the Upper Rhine Valley^19^. At the beginning of the LIA prior to the late sixteenth century, a cluster of years with extremely low GWLs occurred. The long absence of high GWL extremes during almost the entire LIA is also a remarkable feature. Reconstructed GWLs increased by the beginning of the twentieth century in tandem with increasing temperatures (Fig. 3B,C), although large volumes of groundwater resources are increasingly consumed in industrial processes, agricultural production, and domestic use^20^. Furthermore, the increased growth rates observed in living oak trees are positively influenced by human activity, such as modern forest management practices, increased atmospheric carbon dioxide emissions (CO_2_) and levels of nitrogen fertilization into the biosphere^21^. For this reason, a horizontal splitting of the dataset into subsets and the growth level adjustment approach of these subsets after detrending (see “Methods”) is indispensable to avoid an overestimation of reconstructed GWLs in modern times. Precipitation totals and meteorological drought indices derived from instrumental data since the start of the twentieth century show a slight increase in precipitation and tendencies towards generally wetter conditions benefiting oak growth in the region (Supplementary Fig. S6). The observed higher rainfall amounts are likely driven by increasing atmospheric water vapor in the lower atmosphere due to increasing temperatures^22^. Additionally, the GWL of the Upper Rhine catchment (218.300 km^2^, Fig. 1A) is determined by its tributaries, especially the river Aare, which is the most important (560 m^3^/s), contributing significantly to groundwater recharge in the region. Both the Aare and the Rhine River are primarily fed by the Alps, its glaciers and peak summer precipitation, giving the Upper Rhine flow regime an alpine character^23^. Here, the ongoing melting of the Alpine glaciers^24^ with enhanced runoff feeds the Rhine catchment, with a positive influence on the GWL since the late nineteenth century CE.

Since the early 1990s, we observe a divergence between summer temperature and annual mean GWL (Fig. 3C). The GWLs remained stable, whereas the temperature increased rapidly. This decoupling may be either a result of anthropogenically forced unprecedented temperature levels, altering the temperature–groundwater relationship of the past two millennia, through increasing summer evapotranspiration or increasing human use of groundwater reserves or any combination thereof. It should be noted, however, that similar divergences between summer temperature and the annual mean GWL have also occurred in the past. It may thus be an intrinsic part of the non-linear temperature–hydroclimate system. Notable earlier periods of divergence occurred in the mid-sixth, mid-fourteenth and mid-sixteenth centuries CE (Fig. 3C). Nevertheless, considering the past ca. 1700 years, only a few and relatively short periods of decoupling between the temperature and hydroclimate systems were observed, with the most striking period occurring during the LALIA. Here, the GWLs remained stable during extreme and sudden cooling in the decades following 536 CE but decreased together with temperature during the second phase of LALIA cooling at approximately 600 CE. Interestingly, when consulting the speleothem record^5^ from NW Scotland as an independent hydroclimate proxy, both the GWL reconstruction and the speleothem record are well in accordance during the LALIA, and only from 660 CE onwards does a distinct decoupling occur, lasting ca. 40 years (Fig. 3D). Only two other periods, mid-seventeenth and mid-twentieth century CE, show distinct inverse relationships between the two records. Similar to the Alpine summer temperature reconstruction, non-linear responses are only of short-term nature (ca. 50 years), and an overall co-variation between the two records in the low-frequency domain (265–2017 CE *r* = 0.3 and 265–1900 CE *r* = 0.5) is found.

**Figure 4:**
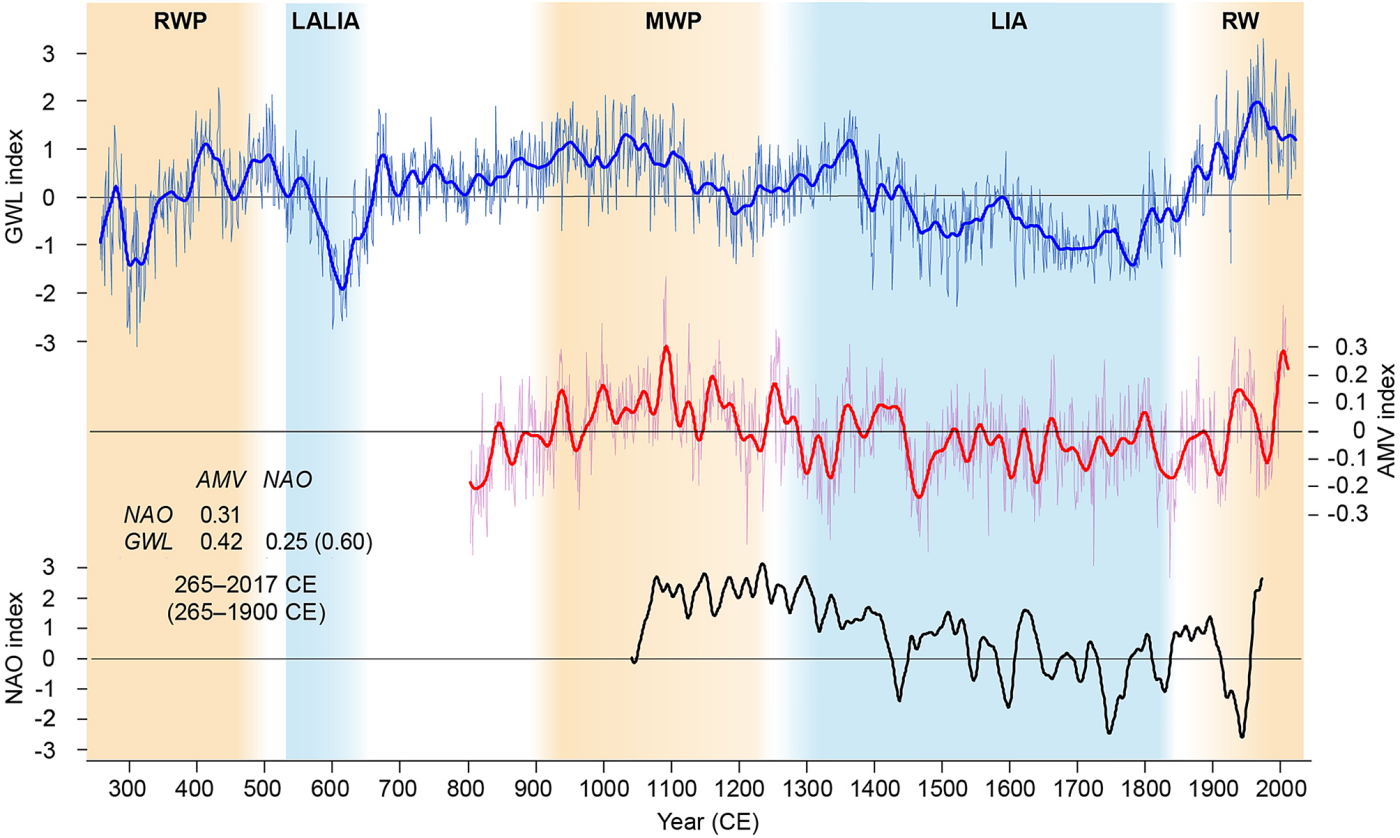
Annual Groundwater level (GWL) reconstruction in comparison with the Atlantic multidecadal variability (AMV)^27^ and the North Atlantic Oscillation (NAO) reconstruction^28^. Bold lines indicate 30 year low-pass filter. Correlation coefficients (*r*) between the reconstructions.

The general agreement between our new annual GWL reconstruction, the Alpine summer temperatures and the hydroclimate conditions across NW Scotland indicates a common driver underlying tree and stalagmite growth in this larger region. The North Atlantic Ocean, as well as the main synoptic mode of atmospheric circulation, the North Atlantic Oscillation (NAO), which also defines summer climate conditions^25^, controls European weather and climate variability^26^. Positive correlation coefficients between the GWL reconstruction, the Atlantic multidecadal variability (AMV)^27^ and the NAO^28^ are found on decadal scales (Fig. 4). A positive relationship between sea surface temperatures of the North Atlantic Ocean and NAO variability at decadal scales has been observed in model simulation runs^29^. Thus, our results suggest that during persistent positive phases of both the NAO and AMV, warm and wet climate conditions prevailed in Western to Central Europe. However, the links between the NAO pattern and European hydroclimate are complex and not yet fully understood. Nevertheless, the few observed decouplings of common warm periods with relatively high GWL during the past 1753 years are found only on decadal and not on centennial time scales (Figs. 3C,D and 4).

In light of current warming, where accelerated hydrological processes presumably cause more and persistent droughts, TRW-based hydroclimate reconstructions are crucial for placing observed hydroclimate changes in a long-term context. These new insights can help to illuminate central issues in human history^6,7^, facilitate future climate predictions and support adaptation and mitigation strategies under contemporary climate change.

## Methods

### Tree-ring data and chronology development

We present a unique compilation of 752 archaeological, subfossil and historical oak (*Quercus spp*.) samples collected at 127 sites along the Upper Rhine Valley (Fig. 1). To allow analyses of growth–climate relationships, we included 285 TRW series of modern oak trees from five sites. The sampling of modern oaks was carried out randomly at three forest stands and two lumberyards within the valley where the historical data were derived. The amount of site control and ecological understanding of the modern samples was designed to be equal to the historical subset; the chronology internal signal-to-noise ratio remains equal throughout time^11,30^.

A total of 1027 TRW series with 50 or more tree rings represent various wood archives and cover the period of 265–2017 CE (Supplementary Figs. S1–S2). We obtained an average annual growth rate of 1.3 and 1.6 mm for the historical material and 2 mm for the recent oaks (Supplementary Fig. S2A). Different growth levels (Supplementary Fig. S2B) and a sharp increase in growth of the modern oaks at the transition from the historical samples from 1850 CE onwards (Supplementary Fig. S2C) required a horizontal splitting of the entire dataset into two historical and a recent subset prior to detrending. To preserve and detect climatic information at different frequencies in the oak TRW chronology, an array of standardization methods was applied to remove biologically induced age trends of the individual TRW series using ARSTAN version 48 (Supplementary Fig. S1)^31^. Finally, one TRW chronology was produced, where the dimensionless indices were computed as ratios after applying a Regional Curve Standardization (RCS)^32^, which retains low-frequency variations in the TRW chronology. A biweight robust mean was calculated after variance stabilization to reduce the effect of varying sample size^33,34^. Due to differences in growth levels (Supplementary Fig. S2), RCS detrending was separately performed for the three subsets (i.e., horizontal splitting). The three subset chronologies (Hist 1, Hist 2, Recent) were scaled to a mean growth of 1.6 mm/year and then averaged to a mean curve. Additionally, changes in variance due to variations in the Rbar values were adjusted using the equation^35^ but with a moving Rbar of 50 years with a 25-year overlap^35^. The chronology was truncated at 265 CE to retain a sample size of equal or larger than seven series.

Sample replication considerably decreased in the first half of the 19^th^ and during the middle of the twelfth century CE, with 22 and 16 series per year, respectively (Supplementary Figs. S1 and S2D). The mean inter-series correlation (Rbar) and the expressed population signal (EPS) statistics^36^, both widely applied measures of internal coherence and replication (EPS > 0.85), demonstrate the high quality of the raw and two detrended composite chronologies over the whole period (Supplementary Fig. S2E).

### Climate and groundwater data

We used monthly gridded climate data from the Climate Research Unit (CRU; TS 4.03) of 0.5° × 0.5° spatial resolution, which were averaged for the region 7°–8.5° E and 47.5°–50° N^37^. Climate variables include temperature, precipitation and standardised precipitation-evapotranspiration index (SPEI)^38^, calculated for an accumulation period of six months (Supplementary Fig. S5). Additionally, we acquired monthly resolved data from nine monitoring wells distributed throughout the research area, where the groundwater table has been continuously measured (meters above sea level) since 1946 (https://www.lubw.baden-wuerttemberg.de, https://www.hlnug.de) (Fig. 1 and Supplementary Fig. S3 and Table S1).

The annual means of the groundwater levels obtained from the nine stations were strongly correlated (mean *r* = 0.52), indicating common annual fluctuations over the entire Upper Rhine Valley (Supplementary Figs. S3). This strong co-variability might reflect a relatively inert, homogenous system controlled by area-wide precipitation and drought, mainly fed by tributaries from the Central Alps.

Spatial correlation maps for the annual GWL and RCS detrended TRW data and hydroclimate variables including precipitation, SPEI-6 and runoff using the CRU TS 4.03 climate grid data (0.5° × 0.5°, p < 0.10) for the period of 1946–2013 were generated using Climate Explorer (https://climexp.knmi.nl).

### Reconstruction

The hydroclimatic reconstruction was developed using a simple scaling technique^39^, which is less susceptible to variance underestimation than are regression techniques^40^. Here, the mean and standard deviation (SD) of the detrended TRW chronology are aligned with the corresponding values of the instrumental climate data, which was done for the groundwater data over the period of 1946–2013 (on anomalies with respect to 1961–90). The robustness of the climate signal was tested for two sub-periods of the calibration period (1946–1979 and 1978–2013). The strength of the relationship between the reconstructed and observed GWL variability was evaluated by the reduction in error (RE)^41^ and coefficient of efficiency (CE)^42^. A skilful reconstruction is obtained when the RE and CE values are positive, which was found using a split calibration-verification technique (i.e., interchanging both sub-periods)^43^. An RE of 0.60 and a CE of 0.16 for the period of 1946–1979 and an RE of 0.64 and a CE of 0.18 for the period of 1980–2013 were obtained for the GWL reconstruction. We used the validation standard error (± 1 root mean square error; RMSE) and the EPS (a maximum of 0.99) to estimate the uncertainty of the reconstructions. Extreme year analysis was conducted on the 30-year high-pass filtered version. A threshold of ± 2 SD was chosen for extreme year identification. The comparison with independent reconstructions was done with normalized data over the common period of 265–2003 CE.

## Supplementary information

Supplementary Information.
