# Supplementary material for: Higher groundwater levels in western Europe characterize warm periods in the Common Era
Source: Sci Rep. 2020 Oct 1;10:16284. doi: 10.1038/s41598-020-73383-8 (PMC7530755; doi:10.1038/s41598-020-73383-8)
Supplement: Supplementary file 1 — Supplementary Information. [file 41598_2020_73383_MOESM1_ESM.pdf]

SUPPLEMENTARY MATERIALS

Higher groundwater levels in western Europe characterize warm periods in the Common Era

Willy Tegel, Andrea Seim, Georgios Skiadaresis, Fredrik Charpentier Ljungqvist, Hans-Peter Kahle, Alexander Land, Bernhard Muigg, Kurt Nicolussi and Ulf Büntgen

Supplementary Figures S1–S10 and Tables S1–S2

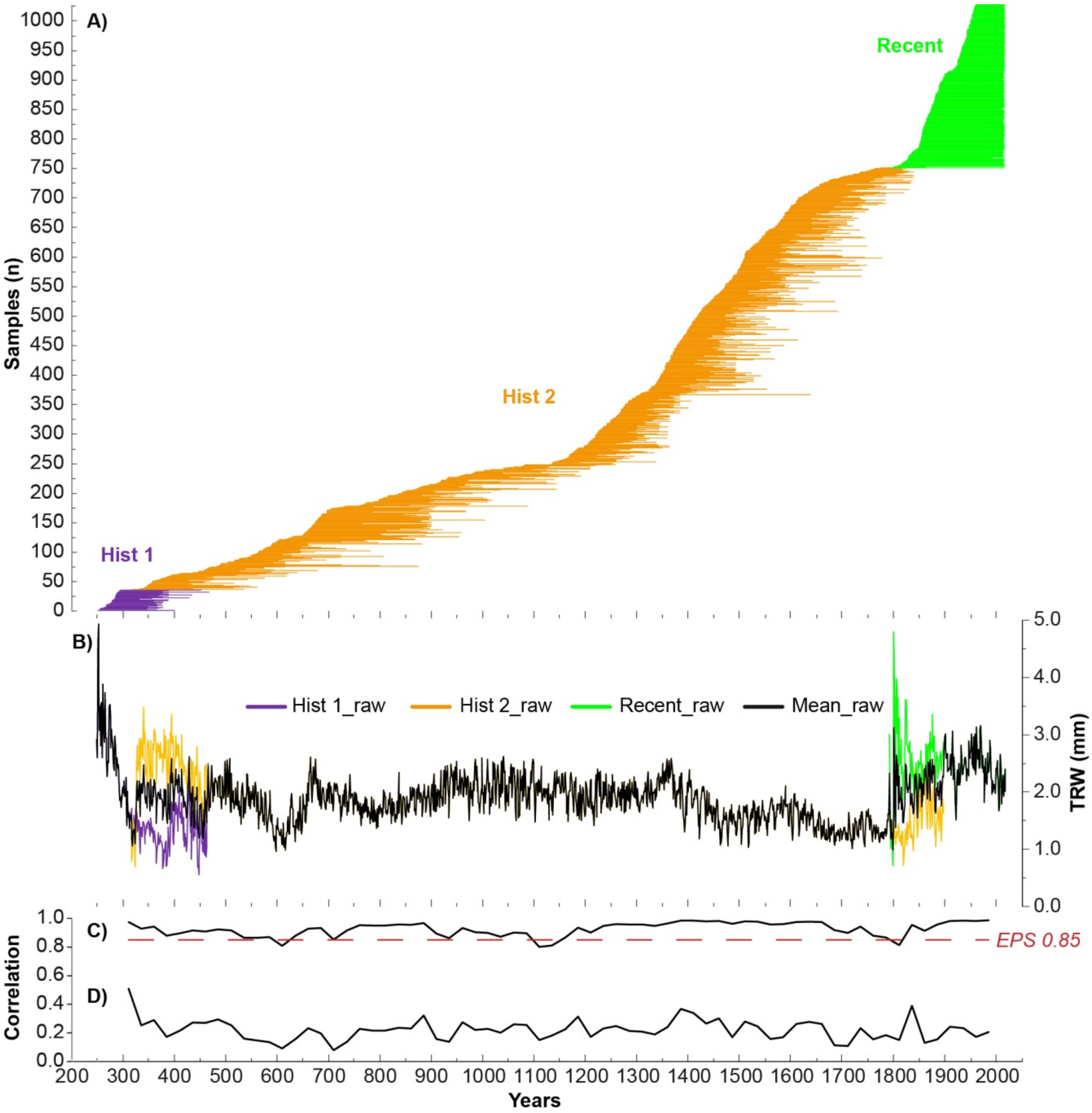

Figure S1. A) Temporal distribution of the 1027 individual oak ring width samples from the Upper Rhine Valley with two historical subsets (Hist 1 and Hist 2; 265–1863 CE) and recent trees (1791–2017 CE), **B)** Raw tree-ring width data for the individual subsets (see legend for colours) and their grant mean (black line) with **C)** the Expressed Population Signal (*EPS*) and **D)** the running inter-series correlation (*Rbar*) of all samples using 50-year windows.

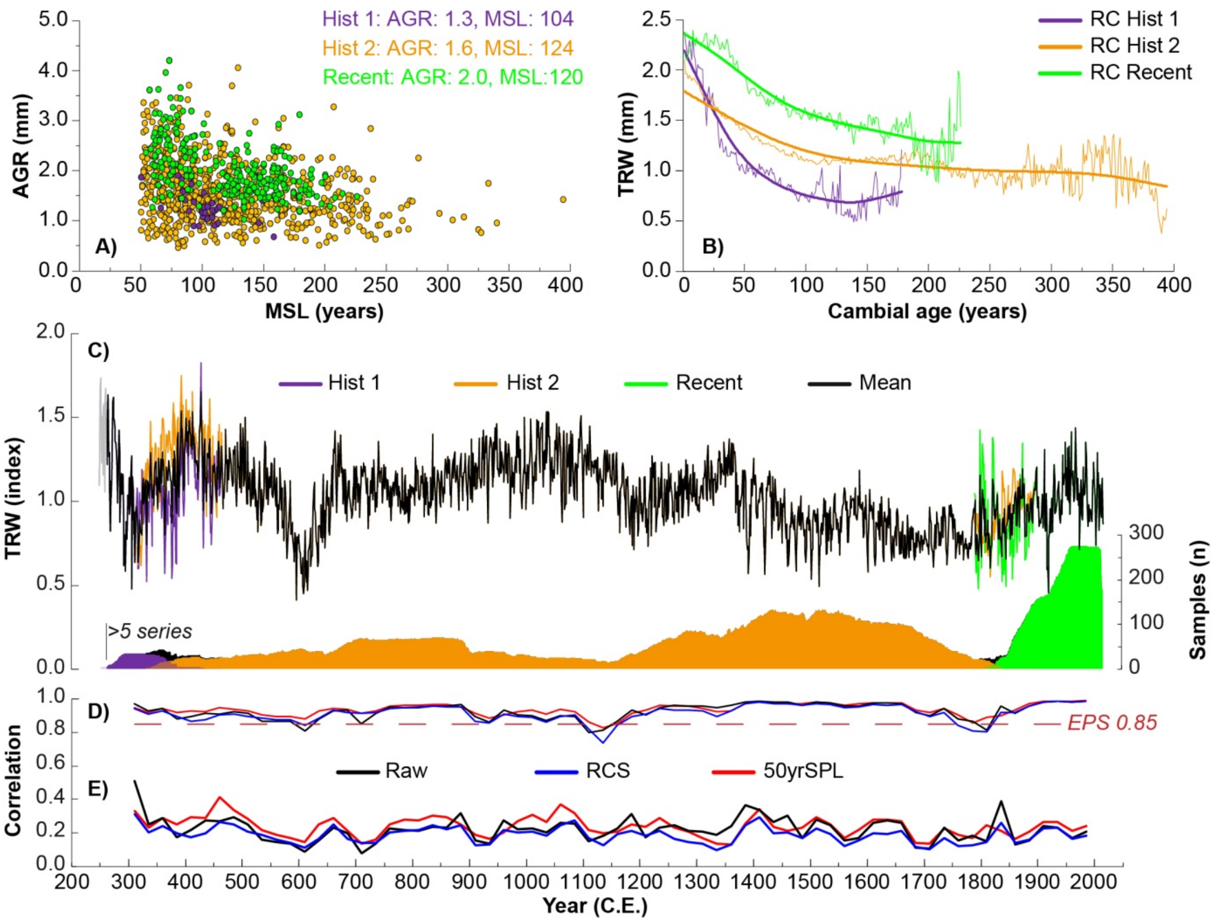

Figure S2. Tree-ring width data characteristics. **A)** Relationship between average growth rates (AGR in mm/year) and mean segment lengths (MSL in years) for each oak sample. **B)** Regional curves (RC) of recent and historical tree-ring data. **C)** Detrended chronology after Regional Curve Standardization (RCS) and sample replication of the two historical subsets (Hist 1 and Hist 2; 265–1863 CE), recent trees (1791–2017 CE) and the combined dataset (265–2017 CE). **D)** Temporal course of the mean expressed population signal (EPS with the 0.85 threshold) and **E)** inter-series correlation (Rbar) using 50-year windows for the raw, RCS, and detrending with cubic smoothing splines with a 50 % frequency cut-off of 50 years (50yrSPL) (see legend for colours).

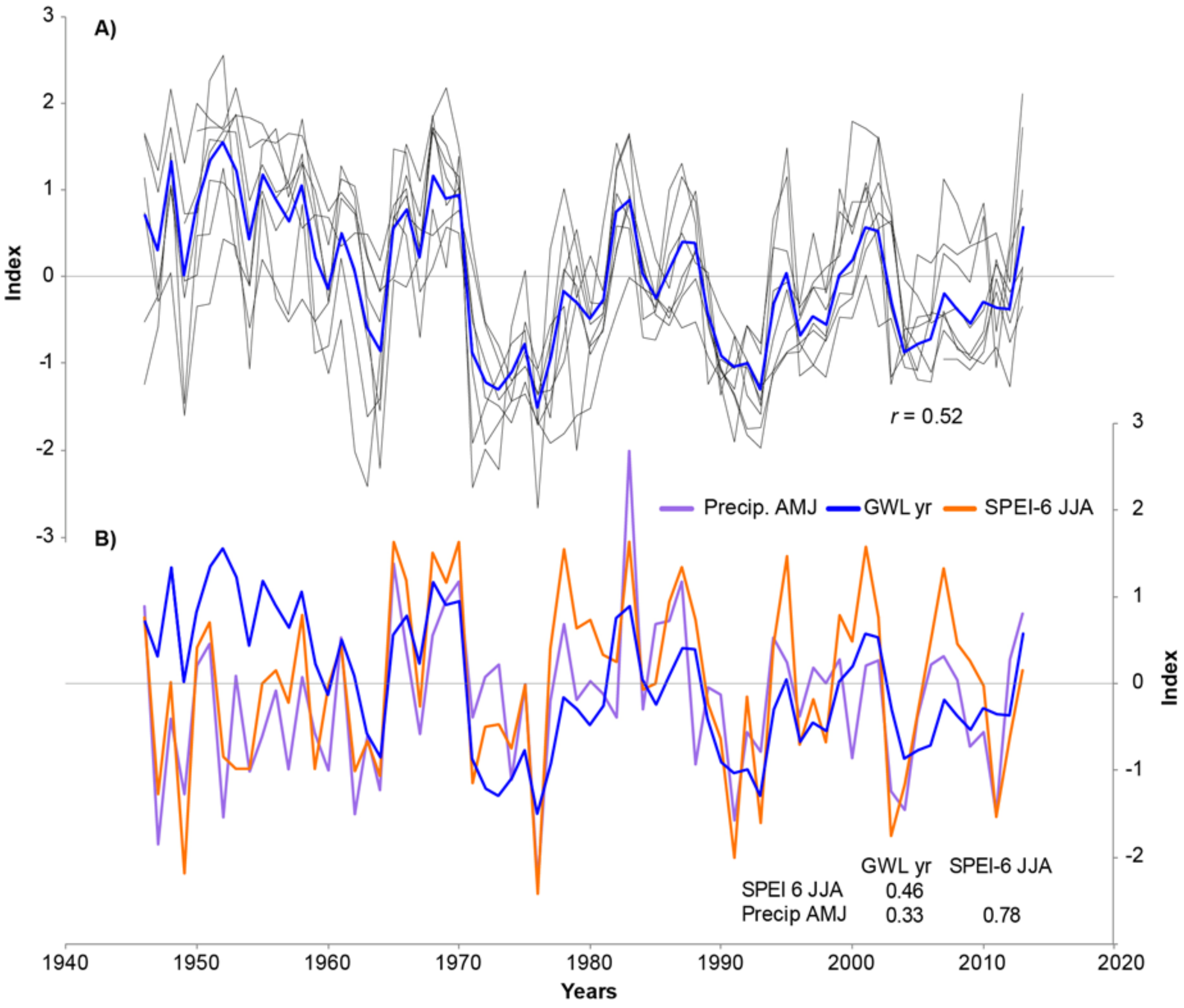

Figure S3. Hydroclimatic instrumental measurements (cf. Tab. S1). **A)** Annual groundwater level variations from nine monitoring wells (Fig.1) and their mean (blue). **B)** June to August drought index (SPEI-6), April to June precipitation totals and annual averaged groundwater level variations for the Upper Rhine Valley (7–8.5°E and 47.5–50°N).

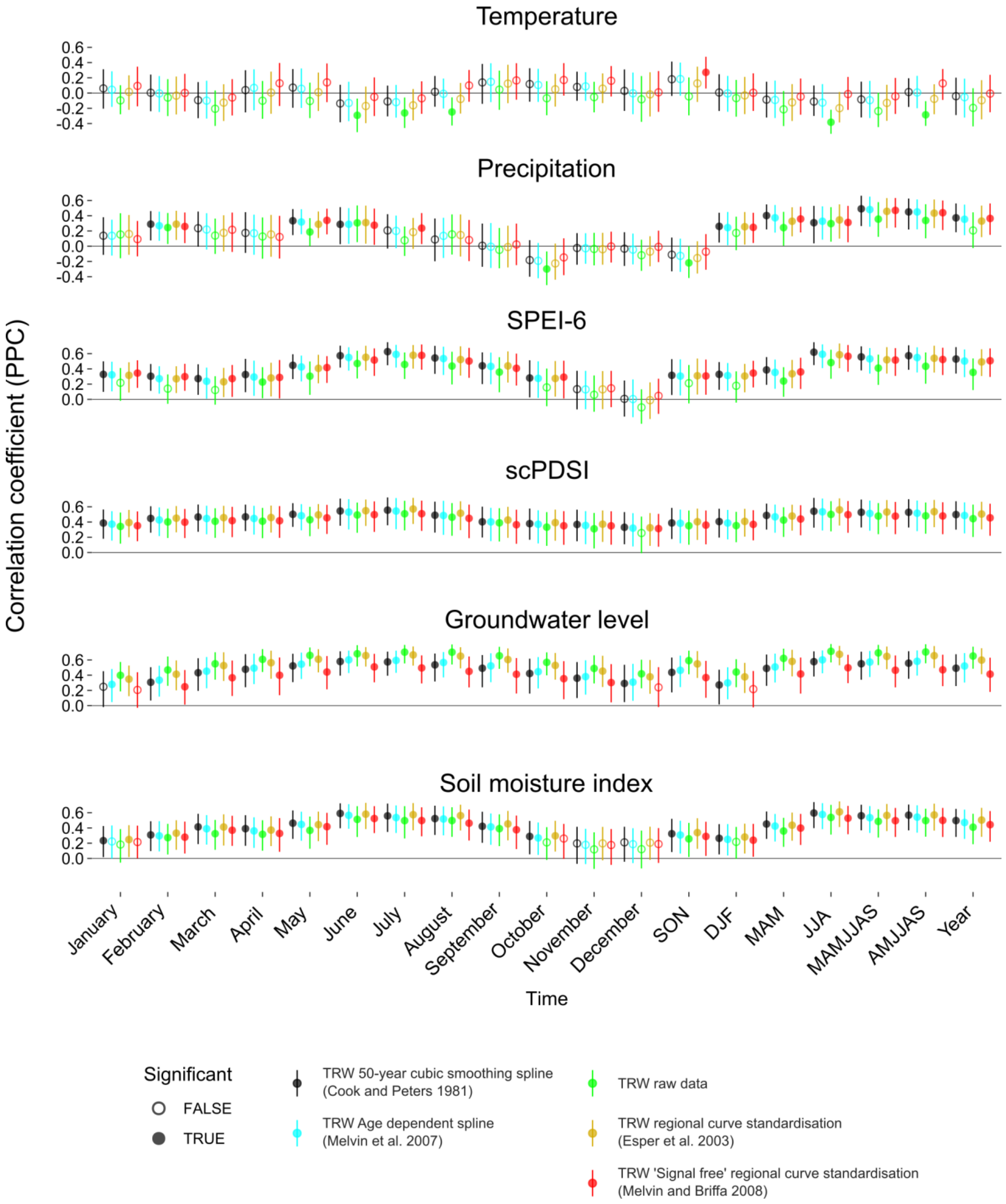

Figure S4. Growth-climate response between the Upper Rhine Valley composite TRW chronology and temperature, precipitation, drought indices (self-calibrated Palmer Drought Severity Index (scPDSI; van der Schrier et al. 2006) and Standardized Precipitation Evapotranspiration Index (SPEI-6; Vicente-Serrano et al. 2010)), groundwater level and soil moisture variations over the 1950–2013 period for each month as well as various seasons. Modelled seasonal soil moisture anomaly information for the total soil column (<180 cm) was obtained from the German Drought Monitor (Samaniego et al. 2013, Zink et al. 2016).

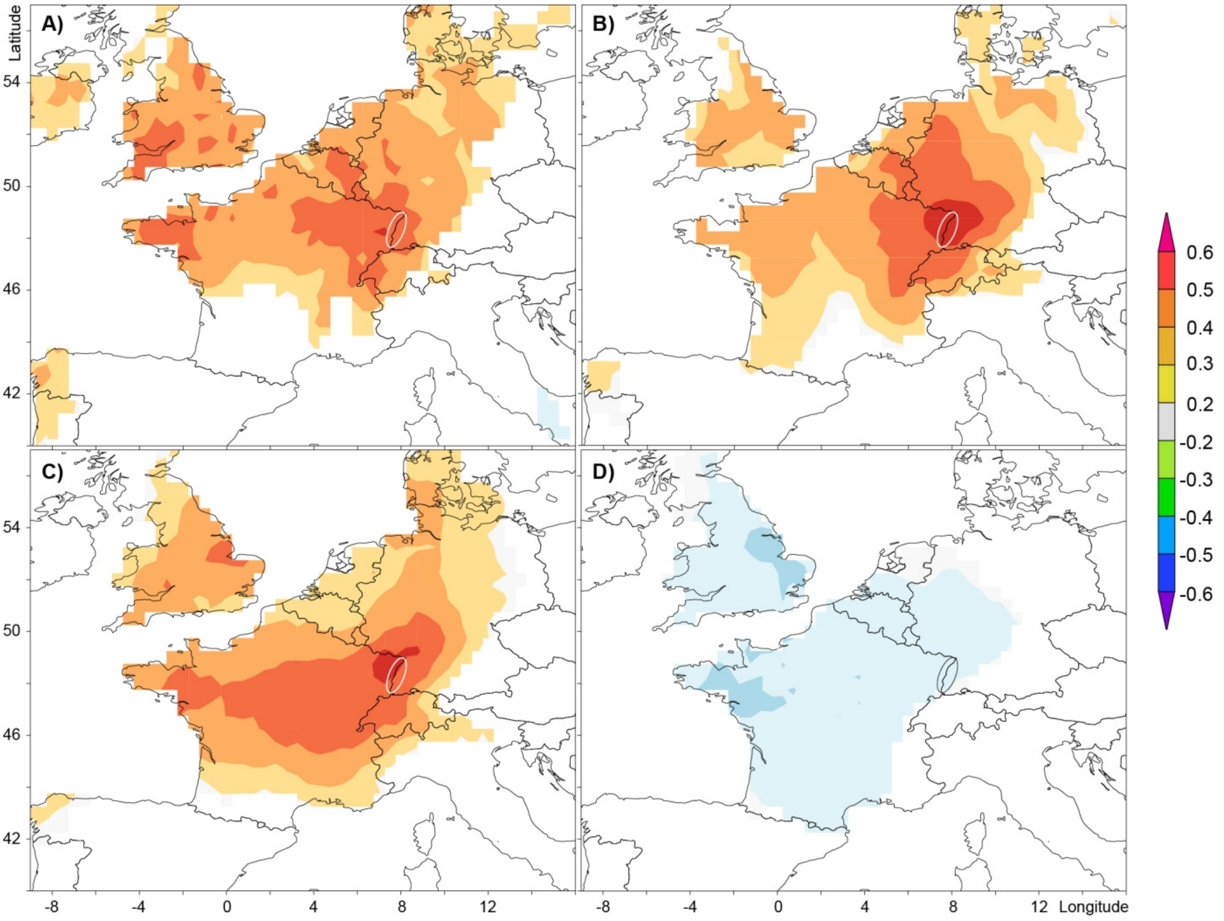

Figure S5. Spatial correlation maps (p<0.10) for the Upper Rhine Valley (ellipse) between the composite TRW chronology after applying a 50-year cubic smoothing spline with 50% frequency cut-off detrending and averaged CRU TS4.03 climate grid data (0.5° x 0.5°) for the June–August season over the 1901–2013 period. **A)** Self-calibrated Palmer Drought Severity Index (scPDSI; van der Schrier et al. 2006) and **B)** Standardized Precipitation Evapotranspiration Index (SPEI-6; Vicente-Serrano et al. 2010), **C)** May–July precipitation and **D)** temperature. All maps were created via the KNMI online server (http://climexp.knmi.nl).

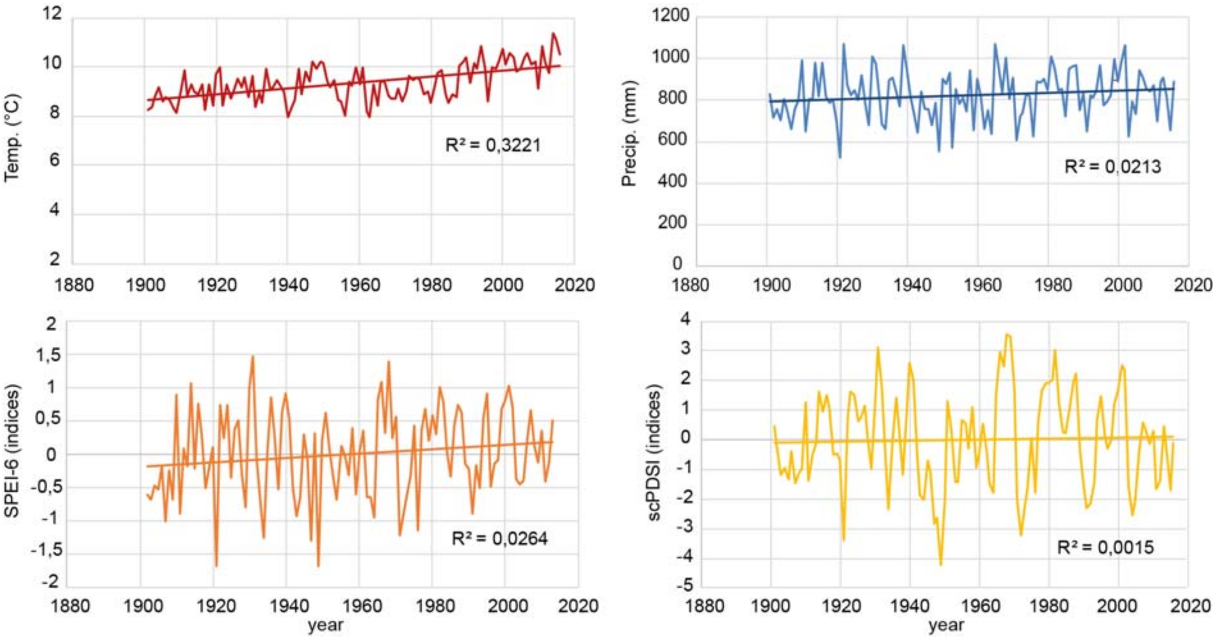

Figure S6. Annual instrumental climate data and indices (CRU TS4.03) averaged over the 7–8.5 °E and 47.5–50°N study region.

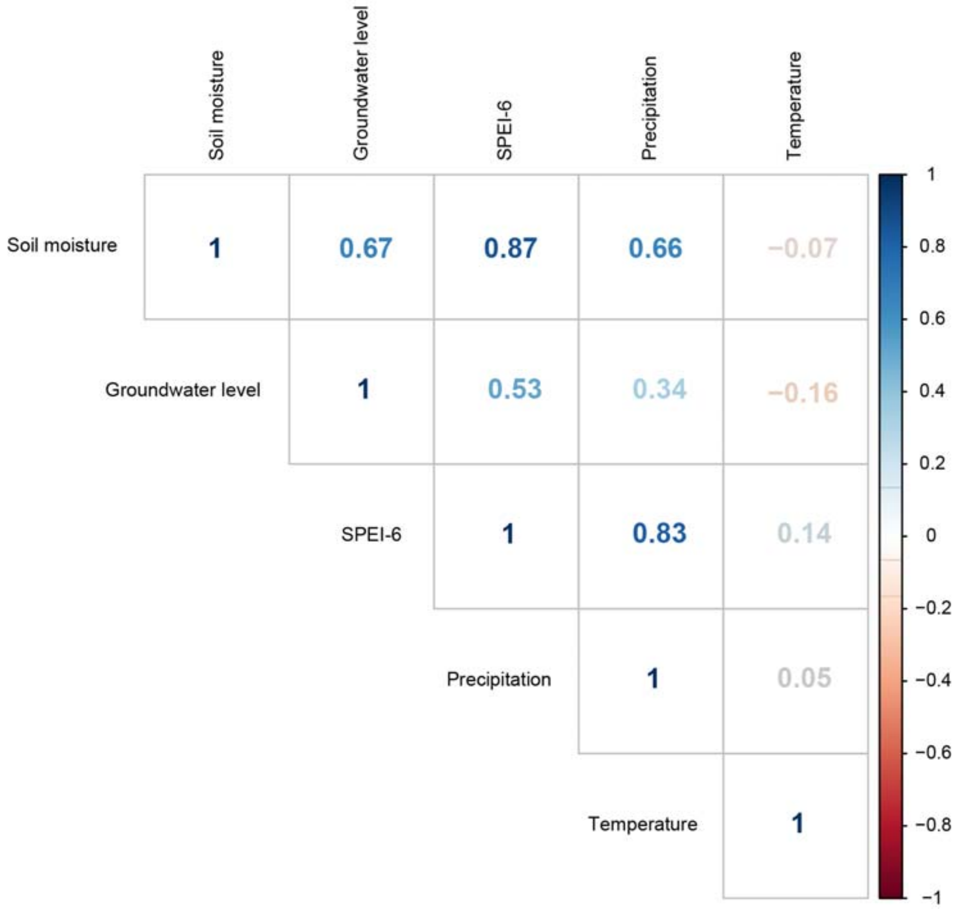

Figure S7. Pearson correlation coefficients (*r*) between annual instrumental climate data and indices (CRU TS4.03) averaged over the 7–8.5 °E and 47.5–50°N study region.

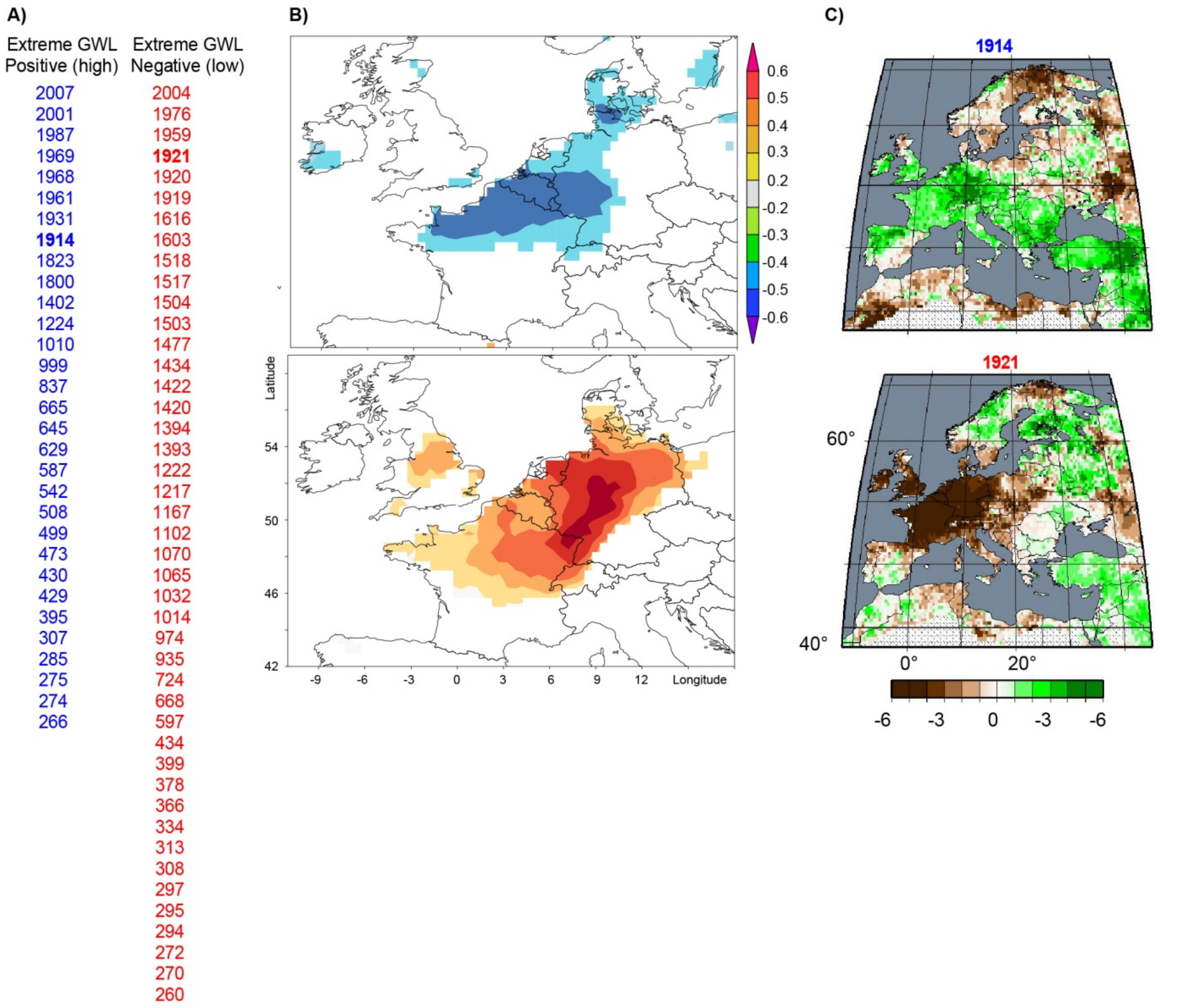

Figure S8. A) Upper Rhine Valley groundwater level (GWL) extremes identified in the high frequency reconstruction (Fig. 3A). Extremes are defined by a standard deviation of ±2. Maximum high GWL was found for 1914 CE, whereas the lowest GWL occurred in 308 and 1921 CE. **B)** Spatial composite maps (http://climexp.knmi.nl) of the 20th century extremes and annual averaged Standardized Precipitation Evapotranspiration Index (SPEI-6, Vicente-Serrano et al. 2010) ($p<0.10$). **C)** Annual maps (0.5° x 0.5° resolution) of June–August hydroclimatic conditions in Europe for 1914 and 1921 CE (OWDA; Cook et al., 2015), respectively. Colours reflect regional hydroclimatic conditions between dryness (brown) and wetness (green).

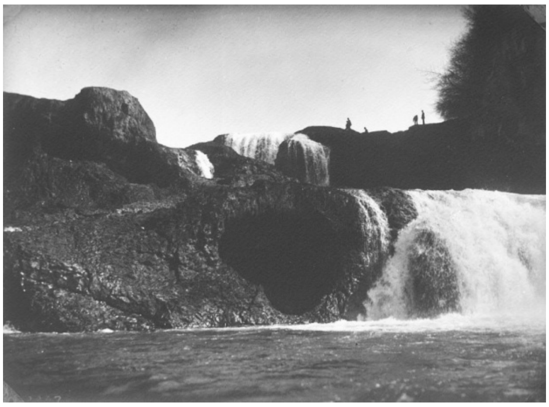

Figure S9. Photograph of the Rhine Falls during the year 1921 showing extreme low water levels. In the lower part of the fall, a large karst cave was visible in the Upper Jurassic limestone. (Stadtarchiv, Nr. J 02.14.01.05/32, CH-Schaffhausen).

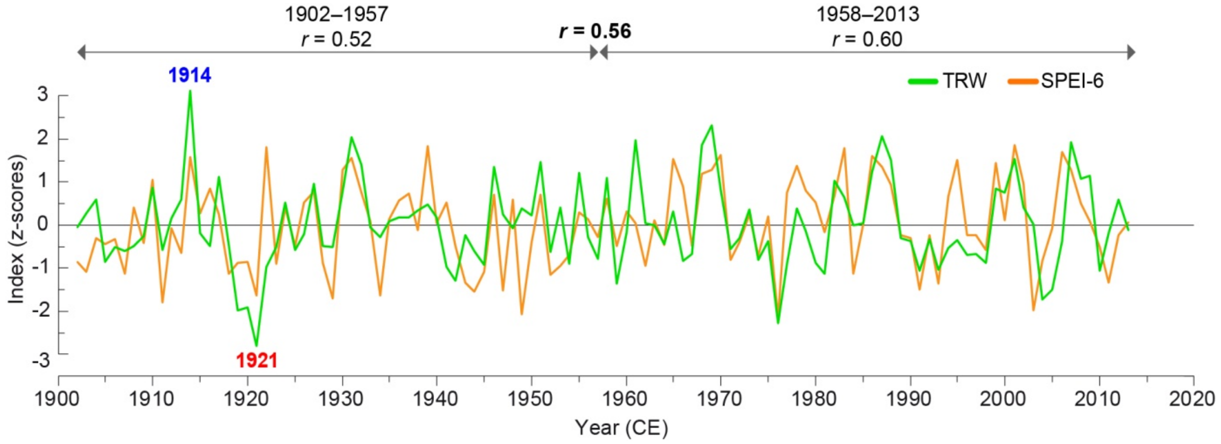

Figure S10. Tree-ring width (TRW) and drought (SPEI-6) relationship. Indexed curves (z-scores) of TRW data after a cubic smoothing spline detrending with a 50 % frequency cut-off of 50 years and the Standardised Precipitation-Evapotranspiration Index (SPEI-6) for June to August over the 1902–2013 period. Correlation results for the periods 1902–1957 (Pearson's correlation coefficients r = 0.51) and 1958–2013 (r = 0.59) are highly significant (P < 0.001). The most extreme years in the 20th century, 1914 and 1921, are highlighted.

Table S1. Groundwater monitoring sites (https://udo.lubw.baden-wuerttemberg.de/ and www.hlnug.de/) with location, site code, latitude (Lat N), longitude (Lat E), country and county.

Location	Site code	Lat N	Lon E	Country	County
Bruchsal	SBR 410	49.140	8.593	Germany	Baden-Württemberg
Weil am Rhein	GWM 1481A	47.627	7.582	Germany	Baden-Württemberg
Offenburg	GWM 984A	48.498	7.921	Germany	Baden-Württemberg
Rheinau (Rastatt)	SBR 657	48.871	8.193	Germany	Baden-Württemberg
Forchheim	GWM 1406A	48.165	7.700	Germany	Baden-Württemberg
Kronau	GWM 341	49.218	8.633	Germany	Baden-Württemberg
Reilingen	GWM SK/3021A	49.275	8.560	Germany	Baden-Württemberg
Sinzheim (Baden-Baden)	GWM 1721	48.764	8.121	Germany	Baden-Württemberg
Viernheim	n° 544271	49.533	8.576	Germany	Hessen

Table S2. Location of the recent oaks with name of the site and coordinates (latitude (Lat N) and longitude (Lon E)). Number of individual tree-ring samples (Series), time period covered (Period), mean series length (MSL; years), average growth rate (AGR; mm) and inter-series correlation (Rbar) as well as the mean sensitivity (Sens), standard deviation (SD) and first-order autocorrelation coefficient (Ac1) are provided.

Location	Lat N	Lon E	Series	Period	MSL	AGR	Rbar	Sens	SD	Ac1
Forst (Submission)	49.162	8.517	44	1811–2015	157	1.6	0.24	0.15	0.34	0.62
Offenburg (Submission)	48.479	7,891	52	1846–2014	135	1.9	0.29	0.16	0.51	0.64
Ottmarsheim (Hardtwald)	47.765	7.457	17	1853–2017	74	2 5	0.42	0.26	1.0	0.66
Freiburg (Mooswald)	48.038	7.843	123	1791–2016	123	2.2	0.28	0.17	0.56	0.55
Lampertheim	49.649	8.543	49	1829–2016	112	1.8	0.29	0.2	0.46	0.51
Oak Network	47.8–49.7	7.5–8.5	285	1791–2017	120	2.0	0.18	0.15	0.44	0.46

References

Cook, E.R., *et al*. Old World megadroughts and pluvials during the Common Era. *Sci. Adv*. **1**, e1500561 (2015).

Cook, E. R. & Peters K. Calculating unbiased tree-ring indices for the study of climatic and environmental change. *Holocene* **7**, 361–370 (1997).

Cook, E. R. & Peters K. The smoothing spline: A new approach to standardizing forest interior tree-ring width series for dendroclimatic studies. *Tree-Ring Bull*. **41**, 45–53 (1981).

Esper, J., Cook, E. R., Krusic, P. J., Peters, K. & Schweingruber, F. H. Tests of the RCS method for preserving low-frequency variability in long tree-ring chronologies. *Tree-Ring Res.* **59**, 81-98 (2003).

Melvin, T.M. & Briffa, K.R. A 'signal-free' approach to dendroclimatic standardisation. *Dendrochronologia* **26,** 71–86 (2008)

Melvin, T.M., Briffa, K.R., Nicolussi, K. & Grabner, M. Time-varying-response smoothing. *Dendrochronologia* **25**, 65–69 (2007).

Samaniego, L., Kumar, R. & Zink, M. Implications of Parameter Uncertainty on Soil Moisture Drought Analysis in Germany. *J. Hydrometeorol.* **14**, 47–68 (2013).

van der Schrier, G., Briffa, K. R., Jones, P. D. & Osborn, T. J. Summer moisture availability across Europe. *J. Clim.* **19**, 2819–2834 (2006).

Vicente-Serrano, S.M., Beguería, S.& López-Moreno, J.I. A Multiscalar drought index sensitive to global warming: the Standardized Precipitation Evapotranspiration Index. *J. Clim*. **23**, 1696-1718 (2010).

Zink, M., *et al*. A. The German drought monitor. *Environ. Res. Lett.* **11**, 74002 (2016).
